# The Intersectoral Coordination Unit for the Sustainable Intensification of Peritoneal Dialysis in Schleswig–Holstein (SKIP-SH) cohort study

**DOI:** 10.1186/s12882-024-03519-9

**Published:** 2024-03-01

**Authors:** Hauke S. Wülfrath, Thorben Schrumpf, Friedrich A. von Samson-Himmelstjerna, Jakob Voran, Yao Zhang, Grit Esser, Sarah-Yasmin Thomsen, Maja L. Messtorff, Theresa Riebeling, Nassim Kakavand, Roland Schmitt, Kevin Schulte, Benedikt Kolbrink

**Affiliations:** 1 Department of Nephrology and Hypertension, University Hospital Schleswig-Holstein (UKSH), Kiel, Germany; 2 Department of Medicine III, Cardiology, University Hospital Schleswig-Holstein (UKSH), Kiel, Germany; 3 German Centre for Cardiovascular Research, Partner Site Hamburg/Kiel/Lübeck, Kiel, Germany

**Keywords:** End-stage renal disease, Kidney replacement therapy, Peritoneal dialysis, Quality of life, Prospective cohort study, Biobanking

## Abstract

**Background:**

Peritoneal dialysis (PD) remains underutilised in Germany, prompting the initiation of the Sustainable Intensification of Peritoneal Dialysis in Schleswig–Holstein (SKIP-SH) project. The SKIP-SH cohort study aims to demonstrate the presumed benefits of PD, including enhanced quality of life and reduced healthcare personnel requirements, and to generate data to strengthen the use of PD.

**Methods:**

The prospective SKIP-SH cohort study recruits patients with advanced chronic kidney disease (CKD) and their caregivers. Comprehensive data, including demographic information, medical history, clinical course, laboratory data, and quality-of-life assessments, are collected. Additionally, biomaterials will be obtained.

Primary study objectives are documenting the clinical course and complications, time on therapy for new dialysis patients, reasons influencing treatment modality choices, circumstances at the initiation of dialysis, and quality of life for patients with CKD and their caregivers. The collected biomaterials will serve as a basis for further translational research. Secondary objectives include identifying factors impacting disease-related quality of life, clinical complications, and therapy dropout, estimating ecological footprints, and evaluating healthcare costs and labour time for initiating and sustaining PD treatment.

**Discussion:**

PD is notably underutilised in Germany. The current therapy approach for advanced CKD often lacks emphasis on patient-focused care and quality-of-life considerations. Furthermore, adequate explorative research programs to improve our knowledge of mechanisms leading to disease progression and therapy failure in PD patients are scarce.

The overarching goal of the SKIP-SH cohort study is to address the notably low PD prevalence in Germany whilst advocating for a shift towards patient-focused care, quality-of-life considerations, and robust translational research.

**Trial registration:**

This study was registered with the German trial registry (Deutsches Register klinischer Studien) on November 7, 2023, under trial number DRKS00032983.

## Background

People with advanced chronic kidney disease (CKD) require kidney replacement therapy (KRT) when the vital functions of the diseased kidneys fail. The most common form of KRT, haemodialysis (HD), is typically delivered in specialised facilities (dialysis centres). However, a proportion of affected patients could potentially undergo peritoneal dialysis (PD), which many can perform independently at home and usually need to visit the dialysis centre only monthly or less.

The two dialysis methods are considered equivalent as patients using them have similar life expectancies [1, 2]. However, PD is advantageous for many people from the quality-of-life perspective because independent therapy implementation allows for greater freedom in everyday life [3, 4]. Another advantage of PD is that the procedure is potentially more cost-effective and efficient than HD because less support is required from healthcare professionals, and the costly transportation to and from the dialysis centre can be minimised [5–7].

Due to historically evolved structures, PD is hardly used in Germany, and well over 90% of patients requiring chronic dialysis perform HD. This situation is particularly pronounced in the northernmost German federal state of Schleswig–Holstein. Here, only 4.3% of dialysis patients perform PD, which appears to be severely underused [8, 9]. Due to its many remote regions and islands with low population density, Schleswig–Holstein is highly suitable for using PD to increase the proportion of people treated at home. Expanding PD use would also reduce the number of in-centre HD patients and ease the workload on the already scarce and burdened specialist staff in HD centres. Intensifying PD use in Schleswig–Holstein could improve the quality of life of respective patients and save considerable costs and resources without limiting the quality of patient care and life expectancy.

Unfortunately, PD cannot be performed indefinitely. During the course of PD therapy, patients develop increasing peritoneal fibrosis and inflammation [10, 11]. These processes lead to detrimental changes in peritoneal transport properties and ultimately to technical failure of the procedure, typically after approximately five years, although prospective studies on this matter are scarce [12, 13]. In the worst case, a potentially fatal condition known as “encapsulating peritoneal sclerosis” develops [14, 15]. The condition shows great variability in between different countries, but in recent years there has been a decline in the incidence. This decline has been attributed to newly developed PD solutions and treatment strategies [16, 17]. Demonstrating the capability to influence one of the most feared complications of PD shows the immense potential and need for future translational research in this area.

**Fig. 1:**
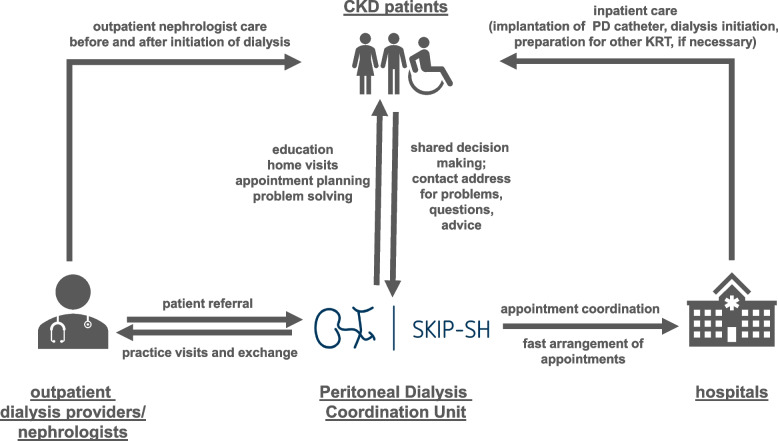
Schematic representation of the Peritoneal Dialysis Coordination Unit functions as an interface between patients and outpatient and inpatient care facilities (CKD = chronic kidney disease, PD = peritoneal dialysis, KRT = kidney replacement therapy, SKIP-SH = Intersectoral Coordination Unit for the Sustainable Intensification of Peritoneal Dialysis in Schleswig–Holstein)

Currently, high structural barriers restrict the further spread of PD. There is a lack of established treatment pathways and the necessary technical expertise due to the low prevalence of PD in many places. To address this problem, we launched *The Intersectoral Coordination Unit for the Sustainable Intensification of Peritoneal Dialysis in Schleswig–Holstein* (SKIP-SH) project at our clinic (Fig. 1). SKIP-SH constitutes a comprehensive initiative designed to augment the prevalence of PD among patients in the region. At its core, this project entails the establishment of a pre-dialysis coordination office to optimise shared decision-making with patients towards the most appropriate dialysis modality and mitigate existing institutional barriers that impede PD adoption. Additionally, SKIP-SH aims to create a training and education structure for patients and medical staff, creating a robust framework for sustainable PD implementation in Schleswig–Holstein. The SKIP-SH cohort study presented here aims to follow the SKIP-SH project and provide data-based proof for the assumed benefits of increased PD use.

## Methods/Design

### Study aims and design

The SKIP-SH cohort study was designed as an accompanying prospective registry study to the clinical SKIP-SH project, which aims to sustainably strengthen PD use in Schleswig–Holstein.

This study aims to determine whether the theoretical benefits of PD are evident in practice and encourage translational research on PD. To do so, we have defined primary and secondary objectives as listed in Table 1.

**Table 1:** Study objectives

Primary objectives	Secondary objectives
Documentation of clinical course, complications, and time on therapy of new dialysis patients	Identification of factors influencing good or poor disease-related quality of life of PD patients
Documentation of the reasons for choosing the treatment modality and the circumstances at the start of dialysis	Identification of factors influencing occurrence of clinical complications and dropout from therapy in PD patients
Documentation of quality of life of patients with CKD starting dialysis and their caregivers before, during, and after dialysis treatment initiation	Estimation of the ‘ecological footprint’ of PD patients
Establishment of a biomaterial bank of patients with CKD starting dialysis	Estimation of health care costs and labour time required to start and maintain PD therapy

### Participant characteristics and inclusion and exclusion criteria

All patients presenting at our pre-dialysis coordination office with CKD stage 4 or 5 (estimated glomerular filtration rate < 30 mL/min/1.73 m^2^ for at least three months)[18] and their family caregiver (living in the same household) are eligible for inclusion in the study. Family caregivers will only undergo the quality-of-life assessment part of the study. All the inclusion and exclusion criteria are summarised in Table 2.

**Table 2:** Inclusion and exclusion criteria

Inclusion criteria	Exclusion criterion
Patients with CKD stage 4 or 5	Inability to give informed consent
Family caregiver of a patient with advanced CKD	
Age ≥ 18 years	

Recruitment started on 1 December 2023 and is scheduled to run for three years; the follow-up will continue at least until 30 November 2028.

In line with the trend of patient numbers that we provided with a permanent dialysis access at our centre annually in recent years, we estimate that ~ 100 eligible patients with CKD qualifying for potential inclusion in the study will present to our pre-dialysis coordination office every year. We expect that the majority of these patients will start a dialysis procedure during the observation period. Patients will be followed up regardless of the dialysis modality they will eventually choose (PD or HD). We expect to include half as many family caregivers as patients.

### Processes, interventions, and comparisons

#### Procedure for informing and obtaining consent

Study information will be provided and written informed consent for participation will be obtained during a meeting between one of the investigators and eligible patients and/or their caregivers at our pre-dialysis coordination office.

#### Data sources

All relevant clinical information and biomaterials will be obtained from the study participants, medical records in our clinic, and the treating physicians in outpatient offices. Quality of life data will be assessed with questionnaires (delivered either in person, by mail, or in a digital form) at predefined time points (applies to patients and caregivers).

#### Data and materials to be collected

The collected dataset will include demographic parameters, clinical trajectory data, laboratory values, biomaterials, quality-of-life assessments, housing conditions, and essential working hours for medical personnel (Table 3).

**Table 3:** Specific data to be collected

**Category**	**Detailed data**
Demographic data	Age; sex; marital status; ethnicity; weight; height; housing details (number of persons in the household, living space in square meters, rent/ownership); care level (if applicable)
Medical history	Comorbidities; current medication use; circumstances of and preparation for starting of dialysis; current dialysis regimen (including number of weekly sessions, Kt/V, dialysate composition and volume, incremental dialysis regimens, if applicable); residual urine excretion (if applicable); reasons for choice of dialysis type (personal preference/medical indications)
Clinical course	Number, duration, and reason for hospital admissions since start of dialysis/in the last year/since the last visit; frequency of outpatient medical consultations and laboratory checks; complications associated with the dialysis procedure; cardiovascular complications; need for care/increase of care level; date and cause of death (if applicable)
Estimated working time of medical staff in association with dialysis treatment	Duration of medical and nursing consultations; time required for learning the dialysis procedure
Laboratory data (as available from routine healthcare)	Blood count; electrolytes; clinical chemistry; lipid status; urinalysis
Quality of life	‘Kidney Disease Quality of Life 36-Item Short Form Survey (KDQOL-36)’ for patients and ‘Burden Scale for Family Caregivers (BSFC)’ for family caregivers
Biomaterials	Whole blood (15 mL per timepoint) and overnight peritoneal effluent

Only basic demographic data and quality of life will be collected from family caregivers; the remaining items will be collected only from the patients.

Table 4 delineates the designated time points for study visits and data/biomaterial collection. Study visits that do not require sampling of biomaterials may be completed via remote communication.

**Table 4:** Data acquisition time points

Time point	First presentation (V0)	Start of dialysis/change of dialysis modality^a^ (V1)	+ 3 months^a, b^ (V1.2)	+ 6 months^a, b^ (V1.3)	+ 12 months (V2)	Annually thereafter (V3, V4, V5, etc.)
Demographic data	x					
Medical history	x	x	x	x	x	x
Clinical course	x	x	x	x	x	x
Estimated working time		x	x	x	x	x
Laboratory data	x	x	x	x	x	x
Quality of life	x	x	x	x	x	x
Biomaterials	x	x			x	x

^a^If the first presentation takes place after the start of dialysis and no change in dialysis type occurs, visits V1-V1.3 will be skipped

^b^May be completed via remote communication

The biomaterials obtained will include blood samples and overnight PD effluent. Samples will be stored to allow for analyses of the transcriptome, DNA(-methylation)-profiling, immunophenotyping of blood leukocytes and cells in the PD effluent, as well as analyses of serum and plasma markers, and soluble markers in the PD effluent.

Digital data documentation and biomaterial storage will be pseudonymised; only the study investigators will have access to the information that enables re-identification.

While data and biomaterials will be stored indefinitely, study participants can revoke their consent at any time and may request the deletion of their data and disposal of their biomaterials.

The publication and provision of data or biomaterials to third parties for scientific purposes will be done exclusively in an anonymised manner.

### Statistical analysis

An overview of the collected data and the study participants will be summarised annually in interim reports during the study period. Exploratory statistical analyses of the study objectives will be conducted as appropriate. This includes an analysis of the correlation between specific characteristics and dialysis regimes on the clinical course and outcomes. As far as feasible, group comparisons will be made between patients on different dialysis regimes, in particular the comparison between PD and HD patients.

Demographic factors and patient characteristics will be compared using the Mann–Whitney *U*-test, Student’s *t*-test, or the Chi-squared test as appropriate. Descriptive analysis will include frequency distribution, medians and interquartile ranges (IQRs), and/or means and 95% confidence intervals. Multivariable regression models will analyse the effects of various variables on outcomes of interest. Time-to-event analyses will be conducted using Kaplan–Meier plots, competing risk analyses, or Cox regression models. Binary variables will be modelled using logistic regression. Statistical significance was set at *p* < 0.05.

## Discussion

Kidney failure disease burden on patients and health care systems is considerable [19] and projected to increase in coming years [20]. Therefore, future increases in patient numbers and dwindling healthcare personnel due to the demographic change could be expected [21]. The landscape of dialysis care in Germany must change fundamentally to overcome these hardships. Innovative strategies such as SKIP-SH are positioned to play a pivotal role in realising these changes, provided they stand on solid scientific footing, which the SKIP-SH cohort study intends to provide.

A comprehensive dataset will be amassed within the study framework, encompassing a spectrum of clinical parameters. This multifaceted dataset will hold significant potential for addressing important scientific questions, thereby contributing substantively to advancing knowledge in the field. We particularly aim to elucidate and address three major issues.

First, the current global dialysis care landscape is characterised by a notable deficiency in home-based approaches [22]. Despite the potential advantages of home dialysis, including improved patient autonomy and quality of life, its prevalence remains disproportionally low [23–25]. Prevailing healthcare infrastructures often favour in-centre HD, perpetuating a systemic preference for facility-based care. In the context of this investigation, our objective is to clarify the factors underlying this phenomenon. Crossing demographic data and housing situations with clinical parameters will allow delving into the potential influence of living conditions on the choice of dialysis modality and treatment adherence, outcomes, and overall patient health after choosing one dialysis modality over another.

Second, clinical trials and day-to-day clinical care often tend toward a disease-oriented paradigm that focuses on pathological aspects rather than a goal-oriented or patient-centred approach. In dialysis care, this tendency might inadvertently result in therapy decisions intransigently prioritising longer life expectancy over quality of life and patients’ personal aspirations. This is especially true for elderly patients and those with multiple comorbidities [26, 27]. Furthermore, clinical course data present an opportunity to delve into the trajectory of disease progression, allowing the discernment of patterns, identification of critical milestones, and assessment of outcomes of certain treatments or interventions. This information is pivotal for refining treatment strategies and optimising patient care. Quality of life assessments provide insights into the holistic impact of dialysis on patients' well-being. By correlating these measures with clinical and demographic data, we could explore the determinants of enhanced quality of life, thereby guiding interventions aimed at improving patient-centred care. This analysis will be conducted on patients and their caregivers. Family caregivers play a pivotal role in treatment outcomes [28, 29]. They also tend to face hardships, which might lead to poor therapy adherence and efficacy [30, 31].

Third, the ongoing demographic change is expected to hit Germany particularly hard over the next decade [32, 33]. With the imminent challenge of more patients and fewer personnel, the incentive to enhance efficiency in healthcare delivery systems becomes paramount [34–36]. It is generally understood that PD at home is more cost-effective than in-centre HD and requires modest personnel input [6, 37]. This necessitates a proactive reassessment of our approach to resource allocation and treatment modalities. Assessment of the working hours required by medical personnel as part of our study will facilitate an evaluation of healthcare system demands and resource utilisation. This information is crucial for optimising staffing levels and improving the efficiency of healthcare delivery, both needed to address the emerging problems affecting dialysis provision in Germany.

Finally, biomaterials collection and subsequent analyses could be put into the context of clinical and laboratory data. This will provide the basis for a deeper understanding of underlying disease mechanisms and could potentially identify specific biomarkers and therapeutic targets in our patient population. This will also enable further research with a translational focus on problems relevant to PD, including peritoneal changes over time or systemic inflammation in the context of cardiovascular complications, aiding the development of future personalised therapeutic approaches [38, 39].

## Data Availability

Data and biomaterials will be shared with researchers who provide a methodically sound proposal. Individual deidentified data collected during the trial will be made accessible. Proposals should be directed to the corresponding author. Data requestors must sign a data access agreement to gain access. Data will be shared to achieve the aims in the approved proposal.

## Abbreviations

CKD: Chronic kidney disease
HD: Haemodialysis
KRT: Kidney replacement therapy
PD: Peritoneal dialysis
SKIP-SH: Intersectoral Coordination Unit for the Sustainable Intensification of Peritoneal Dialysis in Schleswig-Holstein;* Sektorenübergreifende Koordinierungsstelle zur nachhaltigen Intensivierung der Peritonealdialyse in Schleswig-Holstein* [in German]
